# Reciprocal Regulation of C-Maf Tyrosine Phosphorylation by Tec and Ptpn22

**DOI:** 10.1371/journal.pone.0127617

**Published:** 2015-05-20

**Authors:** Chih-Chun Liu, Chen-Yen Lai, Wei-Feng Yen, Yu-Hsien Lin, Hui-Hsin Chang, Tzong-Shyuan Tai, Yu-Jung Lu, Hsiao-Wei Tsao, I-Cheng Ho, Shi-Chuen Miaw

**Affiliations:** 1 Graduate Institute of Immunology, National Taiwan University College of Medicine, Taipei, Taiwan; 2 Division of Rheumatology, Immunology and Allergy, Department of Medicine, Brigham and Women’s Hospital, Harvard Medical School, Boston, Massachusetts, United States of America; University of Tokyo, JAPAN

## Abstract

C-Maf plays an important role in regulating cytokine production in T_H_ cells. Its transactivation of IL-4 is optimized by phosphorylation at Tyr21, Tyr92, and Tyr131. However, the molecular mechanism regulating its tyrosine phosphorylation remains unknown. In this study, we demonstrate that Tec kinase family member Tec, but not Rlk or Itk, is a tyrosine kinase of c-Maf and that Tec enhances c-Maf-dependent IL-4 promoter activity. This effect of Tec is counteracted by Ptpn22, which physically interacts with and facilitates tyrosine dephosphorylation of c-Maf thereby attenuating its transcriptional activity. We further show that phosphorylation of Tyr21/92/131 of c-Maf is also critical for its recruitment to the IL-21 promoter and optimal production of this cytokine by T_H_17 cells. Thus, manipulating tyrosine phosphorylation of c-Maf through its kinases and phosphatases can have significant impact on T_H_ cell-mediated immune responses.

## Introduction

C-Maf is a leucine-zipper transcription factor and plays an important role in T_H_ cells. It contains an N-terminal transactivation domain, which is connected to the C-terminal DNA binding domain through a hinge domain. It is induced by TCR/CD28 and ICOS signals and preferentially expressed in T_H_2, T_H_17, T_FH_ (follicular helper T) and Tr1 [1–4]. It directly transactivates IL-4 and is critical for T_H_2 differentiation [5]. C-Maf also regulates the expansion and maintenance of T_H_17 and T_FH_ cells via inducing IL-21[3]. It acts synergistically with Sox5t to induce RORγt to promote T_H_17 differentiation but negatively regulates the production of IL-22 [6, 7]. Moreover, c-Maf is induced by IL-27 and works cooperatively with aryl hydrocarbon receptor to promote the development of Tr1 cells and their expression of IL-10 [8, 9].

Tyrosine phosphorylation is a critical regulatory process of signal transduction. It controls various cellular events including cell cycle regulation, cell signaling, and protein trafficking. In addition to cytoplasmic signaling molecules, the activity of a handful of transcription factors is also subject to regulation by tyrosine phosphorylation. We have previously shown that c-Maf can be phosphorylated at Tyr21/92/131 in T_H_2 cells. Tyrosine phosphorylation is critical for the recruitment of c-Maf to the IL-4 promoter and the optimal production of IL-4. In addition, T_H_ cells from glycemic NOD mice display attenuated tyrosine phosphorylation of c-Maf compared to those of euglycemic NOD mice [10]. Despite these observations, it is unclear how tyrosine phosphorylation of c-Maf is regulated in T_H_ cells and whether this process also plays a role in other T_H_ subsets.

Three Tec kinases, Itk, Rlk and Tec, are highly expressed in T cells and their activity is induced upon antigen engagement [11–13]. Moreover, Tec kinases are differentially expressed in different T_H_ subsets and play an important role in regulating the function and differentiation of T_H_ cells [14]. However, there probably exists functional redundancy among these three Tec kinases. For example, Rlk-deficient mice display a normal T_H_1 cytokine profile and marginal defect in T_H_1 response against *T*. *gondii* infection [15, 16]; further, deficiency of Tec has minimal impact on the differentiation of T_H_1 and T_H_2 cells [17]. One of the substrates of Tec kinases is T-bet, a transcription factor that is essential for the differentiation of T_H_1 cells. T-bet can be phosphorylated at Tyr525 by Itk [18]. The tyrosyl phosphorylated T-bet interacts with GATA-3, preventing GATA-3 from binding to IL-4 promoter [18]. It is still unclear whether Tec kinases also act on other transcription factors in T_H_ cells.

Ptpn22, a member of non-transmembrane type protein tyrosine phosphatases (NT-PTPs), is expressed mainly in hematopoietic cells [19]. One of its known functions is damping activation signals in lymphocytes via its interaction with various cytoplasmic signaling molecules, including LCK CSK,VAV, and ZAP70 [20–22]. Accordingly, deficiency of Ptpn22 leads to abnormal expansion of memory/effector T cells and increased antibody production [23]. Genome-wide association studies have identified a missense single nucleotide polymorphism of Ptpn22 that is strongly associated with higher risk of several autoimmune diseases, including rheumatoid arthritis and SLE [24–26]. Although Ptpn22 was originally identified as a cytoplasmic protein, it actually contains a nuclear localization signal and is present in the nucleus of macrophages [27]. We have demonstrated that nuclear Ptpn22 is functionally distinct from cytoplasmic Ptpn22 [27]. However, it is still unknown as to whether Ptpn22 is also present in the nucleus of T cells and, if it is, what the role of nuclear Ptpn22 is in T cells.

Here, we show that Tec, but not Rlk or Itk, is a tyrosine kinase of c-Maf. We further show that Ptpn22 is also present in the nucleus of T_H_ cells and directly interacts with c-Maf. It counteracts the effect of Tec and dephosphorylates c-Maf. Furthermore, phosphorylation of c-Maf at Try21/92/131 is also critical for optimal expression of IL-21 in T_H_17 cells. Our results uncover novel ways to manipulate the status of tyrosine phosphorylation and subsequently the activity of c-Maf.

## Materials and Methods

### Mice

Ptpn22-deficient (Ptpn22 KO) mice in C57BL/6 background have been previously described [23]. C57BL/6 mice were purchased from the Laboratory Animal Center of NTUMC or NAR Labs. All animals were housed under specific pathogen-free conditions. This study was carried out in strict accordance with the recommendations in the Guide for the Care and Use of Laboratory Animals of the National Institutes of Health. The protocol was approved by the Institutional Animal Care and Use Committee (IACUC) of National Taiwan University College of Medicine and College of Public Health (Permit Number: 20100308). Total of 70 C57BL/6 mice (from NTUMC or NAR Labs) and 20 Ptpn22-deficient (Ptpn22 KO) mice (from Harvard University) were used in this study. Water and food were provided sufficiently daily. All mice were killed by CO_2_.

### Primary mouse T_H_ cells and cell lines

CD4^+^ T cells were cultured in complete RPMI 1640 medium [containing 10% FBS, 2 mM L-glutamine, 1 mM sodium pyruvate, 0.1 mM non-essential amino acids, 100 U/ml of penicillin, 100 μg/ml of streptomycin (all from Hyclone), 57 μM β-mercaptoethanol (Sigma-Aldrich) and 10 mM HEPES]. Human embryonic kidney fibroblast cell line (HEK 293T cells; ATCC CRL-11268) and EL4 thymoma cells **(**ATCC TIB-39**)** were cultured in complete DMEM medium (containing 10% FBS, 2 mM L-glutamine, 0.1 mM non-essential amino acids, 100 U/ml of penicillin, 100 μg/ml of streptomycin and 10mM HEPES).

### Plasmids

pCDNA3.1 (Invitrogen) expressing mouse HA-c-Maf, c-Maf Y8F, Y21/92 and Y3F mutants, and pEGFP-c-Maf were previously described [10]. pEGFP-c-Maf domain mutants (AD, HINGE, DBD) were generated by amplifying mouse c-Maf activation domain (AD), HINGE domain (HINGE), DNA binding domain (DBD) domains and cloned into pEGFP-C1 (Clontech). pDsRed2-c-Maf was generated by amplifying mouse c-Maf gene and cloned into pDsRed2-C1 (Clontech) was described previously [28]. pME18S Tec (encoding murine Tec), pME18S Tec KD (encoding kinase-dead Tec protein) and pME18S Itk (encoding murine Itk) were gifts from Dr. Wen-Chin Yang [29, 30]. pCMV-3Tag-3-Tec (encoding mouse Tec-3XFLAG), pCMV-3Tag-3-Tec KD (encoding mouse Tec KD-3XFLAG) and pCMV-3Tag-3-Itk (encoding mouse Itk-3XFLAG) were generated by amplifying mouse cDNA with primers containing EcoRI (forward primer) or XhoI (reverse primer) from pME18S Tec, pME18S Tec KD and pME18S Itk, respectively. The amplified cDNA was cloned in-frame into pCMV-3Tag-3A (Agilent Technologies). pCMV-3Tag-3-Rlk (encoding mouse Rlk-3XFLAG) was generated by amplifying mouse Rlk cDNA with primers containing EcoRI (forward primer) or XhoI (reverse primer). The amplified cDNA was cloned in-frame into pCMV-3Tag-3A (Agilent Technologies). GFPRV Tec was generated by amplifying mouse Tec from pME18S Tec and cloned into GFPRV backbone. LV shTec was derived from pLKO_TRC001 shTec (shRNA 677) obtained from Academic Sinica RNAi CORE, ROC, by replacing puromycin gene with GFP gene. pEYFP-Ptpn22 generated by amplifying mouse Ptpn22 cDNA and cloned in-frame into pEYFP-C2 (Clontech) was previously described [27].

### Antibodies, cytokines and reagents

Anti—c-Maf (M-153), anti-Hsp90 (sc-7947) and anti-Oct1 (sc-232) antibodies were purchased from Santa Cruz Biotechnology (Santa Cruz, CA). HRP-conjugated 4G10 (anti-phosphotyrosine), HRP-conjugated goat anti-mouse and goat anti-rabbit L chain antibodies were purchased from Millipore (Billerica, MA). Anti-p-Tyr (P-Tyr-1000) antibody was purchased from Cell Signaling Technology (Danvers, MA). Anti-HA (12CA5) antibody and Protein A and G agarose beads were purchased from Roche (Mannheim, Germany). Anti-FLAG (M2) antibody was purchased from Sigma-Aldrich (St. Louis, MO). Anti-EGFP (ab290) antibody was purchased from Abcam (Cambridge, MA). Anti-mouse Ptpn22 (anti-PEP) antibody was obtained from Genentech (San Francisco, CA). Anti-Tec antibody was purchased from Upstate biotechnology. Anti-α-Tubulin 4a antibody was purchased from GeneTex. Antibodies to CD3-ε (17A2), CD28 (37.51), IL-4 (11B11), and IFNγ (XMG1.2) were purchased from BioLegend (San Diego, CA). Recombinant human IL-2 and TGF-β and recombinant mouse IL-4 and IL-6 were purchased from PeproTech (Rocky Hill, NJ). Recombinant mouse IL-23 was purchased from eBioscience (San Diego, CA).

### Cell transfection

HEK 293T cells (2–3 x10^6^) were seeded in a 10 cm^2^ culture dish and transiently transfected with JetPEI (Polyplus Transfection, New York, NY) according to the manufacturer’s instructions. EL4 cells (1 x10^7^) were electroporated with plasmids using MicroPorator MP-100 (Digital Bio) according to the manufacturer’s instruction.

### In Vitro T_H_ cell differentiation

CD4^+^ T cells were isolated from lymph nodes and spleens of 5- to 8-week-old C57BL/6 mice by EasySep Mouse CD4 Positive Selection Kit (Stem Cell Technologies, USA). CD4^+^ T cells were seeded at 1x10^6^ cells/ml in complete RPMI 1640 growth medium and stimulated with plate-bound anti-CD3 (1 μg/ml) and soluble anti-CD28 (2 μg/ml) under T_H_2-skewing (10 μg/ml of anti-IFNγ, and 10 ng/ml of IL-4) or T_H_17-skewing (10 μg/ml of anti-IL-4 antibody, 10 μg/ml of anti-IFNγ antibody, 20 ng/ml of IL-6, 20 ng/ml of IL-23, and 2.5 ng/ml of TGF-β) conditions. Under T_H_2-skewed conditions, Human IL-2 (100 U/ml) was added with fresh medium every other day; however, for T_H_17-skewed conditions, Human IL-2 (50 U/ml) was added on day 0 only.

### Retrovirus infection

After 40 hours of stimulation with plate-bound anti-CD3 and soluble anti-CD28 antibodies, CD4^+^ T cells (1 x 10^6^) were mixed with retrovirus and polybrene (8 μg/ml; final concentration) in a 24-well plate. Cells were centrifuged at 2200 r.p.m for 1 hour at 30°C and then incubated at 37°C for 30 min. Viral supernatant was replaced by complete RPMI 1640 growth medium. After additional incubation for 48 hours, infected (GFP^+^) cells were sorted on FACSAria (BD Biosciences, San Jose, CA).

### Immunoprecipitation (IP) and Western blotting (WB)

For detection of tyrosine phosphorylated c-Maf, cells (1x10^7^ cells) were pretreated with 100 μM pervanadate [derived from sodium orthovanadate (Sigma-Aldrich)] for 10 min then lysed in 500 μl RIPA buffer (50 mM Tris-HCl, pH 7.4, 150 mM NaCl, 1% NP40, 0.5% sodium deoxycholate, 0.1% SDS and 1 mM EDTA) containing 1 mM PMSF (Sigma-Aldrich) and complete protease inhibitor mixture [100 μM pervanadate and plus phosphatase inhibitor (Roche)]. Appropriate antibody (≤2 μg) was added to the lysates, which were shaken for 1–3 hours at 4°C. Protein A/G agarose beads (25 μl per tube) were added into antibody-lysate mixture and gently mixed for an additional 2–3 hours at 4°C. The entire mixture was washed three times with cold PBS and analyzed by Western blotting. Cytoplasmic and nuclear extracts were prepared by washing cells with cold PBS and resuspended in hypotonic lysis buffer (10 mM HEPES [pH 7.9], 1 mM MgCl_2_, 10 mM KCl, 0.1% Triton X-100, 20% glycerol, 0.5 mM PMSF, and protease inhibitors) on ice for 10 min. The supernatant, corresponding to the cytoplasmic fraction, was collected by centrifugation at 13,000 X g for 10 min at 4°C. The nuclear pellet was washed with hypotonic lysis buffer and then resuspended in hypertonic lysis buffer (10 mM HEPES [pH 7.9], 400 mM NaCl, 1 m EDTA, 0.1% Triton X-100, 20% glycerol, 1 mM PMSF, and protease inhibitors) and then incubated on ice for 20 min. Nuclear extract was collected by centrifugation. Western blotting was performed according to a previously described protocol [10]. Densitometry readings of Western blots were obtained and analyzed with ImageJ program. Degree of c-Maf tyrosine phosphorylation was calculated by dividing the density of phosphorylated c-Maf with the density of total c-Maf.

### Luciferase assay

HEK 293T cells (1x10^5^) were co-transfected with pGL3-*Il4*-Luc reporter, Renilla reporter plasmid (pTK-RL) and various expression vectors. Twenty-four hours after incubation at 37°C, cells were lysed and analyzed by using the Dual-Glo Luciferase Assay System (Promega, Madison, WI) according to the manufacturer’s instructions. Firefly luciferase activities were normalized against Renilla luciferase levels.

### Measurement of IL-4, IL-17 and IL-21 production in primary T cells by ELISA

CD4^+^ T cells (1 x 10^6^/ml) were cultured with complete RPMI 1640 growth medium and stimulated with plated-bound anti-CD3 (1 μg/ml) for 24 hours. The supernatant of the culture medium was collected and the concentration of IL-4, IL-17 and IL-21 was measured using Ready-Set-Go kit (eBioscience), according to the manufacturer’s instruction.

### Confocal microscopy analysis

HEK 293T Cells (4x10^5^ cells/well) were cultured on glass cover slips in six-well culture plates containing 10% FBS DMEM for 24 hours and were transfected with vectors expressing DsRed2-c-Maf or DsRed and EYFP-Ptpn22 or EYFP. Twenty-four hours after transfection, cells were fixed, stained with DAPI, and then analyzed with a Leica confocal microscope (Leica TCS SP5, 63X (oil) objective lens).

### Chromatin immunoprecipitation (ChIP)

ChIP was performed according to a published protocol [10]. Briefly, T_H_ cells (1x10^7^) were fixed with 1% formaldehyde. Chromatin was sheared by sonication to 200 to 600 base pairs in size. Anti-FLAG antibody (M2; Sigma-Aldrich) and normal mouse IgG (Santa Cruz Biotechnology) were used for immunoprecipitation. The precipitated DNA was analyzed by quantitative PCR using primers corresponding to mouse *Il21* promoter and the data are presented as percentage of input DNA. The following primers were used: *Il21* promoter forward primer: 5’-TGGTGAATGCTGAAAACTGGA-3’; *Il21* promoter reverse primer: 5’-CTAGGTGTACGTGTGCGTGT-3’

### Yeast two-hybrid screening

Yeasts (EGY48) were grown in YPAD (1% yeast extract, 2% peptone, 2% glucose and adenine). The yeasts were co-transformed with bait plasmid (pGilda-cMaf-HINGE, encoding DNA binding domain of LexA and hinge domain of c-Maf chimeric protein), prey plasmid (pJG4-5-cDNA library from an activated murine CD8^+^ T cell line, L3 cell line) and LacZ reporter pSH18-34 (Invitrogen). The transformed yeasts were selected by synthetic media (0.67% yeast nitrogen base without amino acids, 2% glucose, auxotrophic amino acids). Yeast colonies (3.6x10^6^) were screened. Positive yeast clones were obtained by survival test and X-gal analysis. Prey plasmids encoded potential c-Maf-interacting proteins were isolated and sequenced.

### Statistical analysis

Statistical analyses were performed with unpaired, two-tailed Student’s *t*-tests unless stated otherwise. A value of *P*<0.05 was considered statistically significant.

## Results

### Tec is a tyrosine kinase of c-Maf

**Fig 1:**
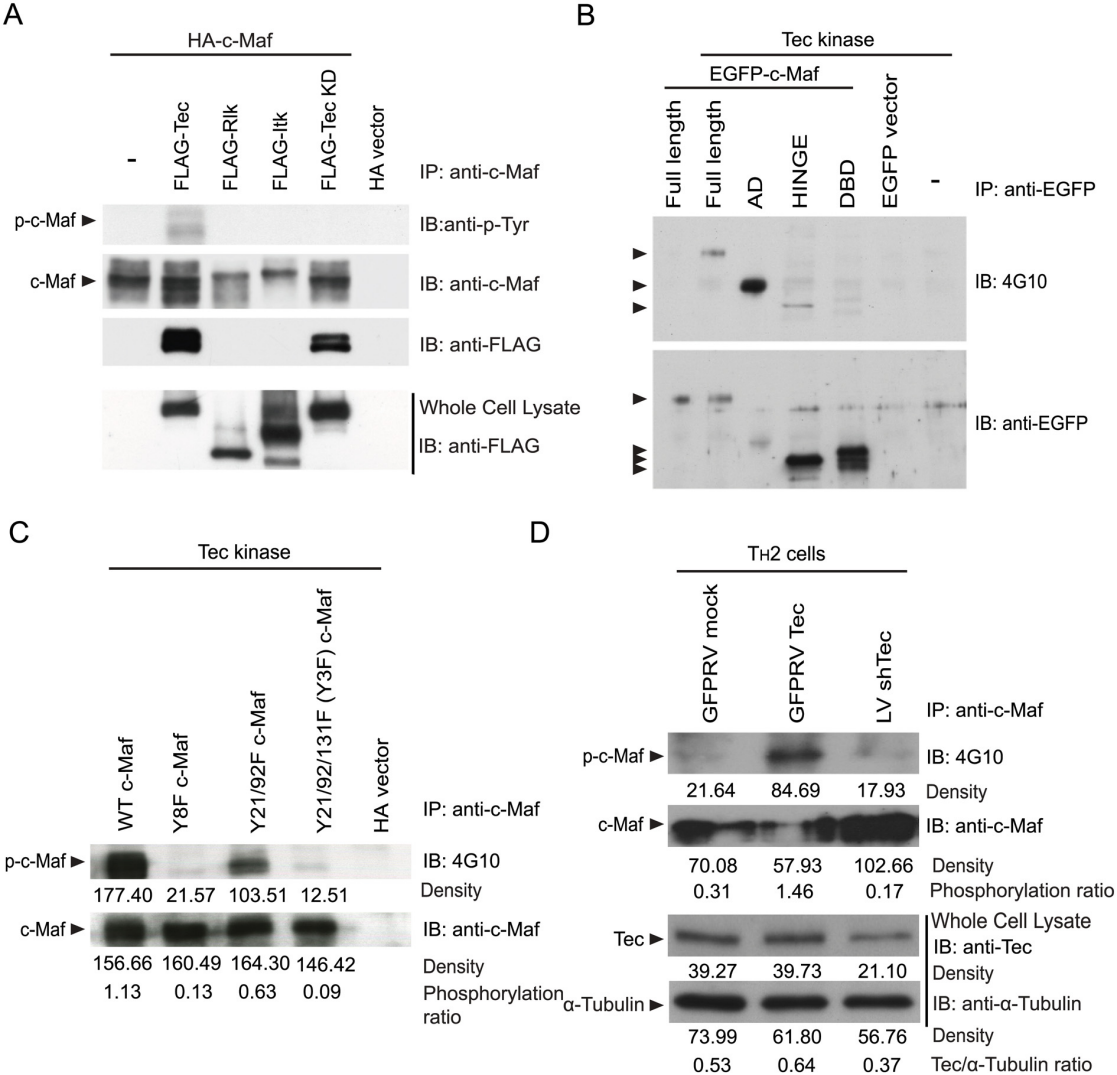
Tec induces tyrosine phosphorylation of c-Maf. (**A**) HA-c-Maf was co-expressed with various C-terminal 3XFLAG-tagged Tec kinase members (Tec, Rlk or Itk) or a kinase dead Tec (Tec KD) in HEK 293T cells. Whole cell extract was harvested after 24 hours and immuno-precipitated (IP) with anti-c-Maf antibody. The immunoprecipitant was then immuno-blotted (IB) with anti-p-Tyr, anti-c-Maf and anti-FLAG antibodies. (**B**) Tec was co-expressed with EGFP-fused full-length, activation domain (AD), HINGE domain (HINGE), or DNA binding domain (DBD) of c-Maf in EL4 cells. The cells were lysed 24 hours after transfection and cell extract was immunoprecipitated with anti-EGFP antibody. The immunoprecipitant was then probed with 4G10 or anti-EGFP antibody. The various forms of c-Maf were marked with arrowheads. (**C**) Wild type c-Maf or various c-Maf mutants, in which tyrosine residues were converted to phenylalanine, were co-expressed with Tec in EL4 cells. The transfected cells were lysed 24 hours later and then immunoprecipitated with anti-c-Maf antibody. The immunoprecipitant was then probed with 4G10 or anti-c-Maf antibody. Y8F carries Y-to-F mutation at Tyr21/91/92/97/131/181/341/345. Phosphorylation ratios of c-Maf are also shown. (**D**) Naïve T_H_ cells were stimulated under T_H_2 skewing conditions for 48 hours and then transduced with retrovirus expressing GFP alone (GFPRV mock) or along with murine Tec (GFPRV Tec) or lentivirus expressing shRNA specific for Tec (LV shTec). Forty-eight hours later, the transduced cells were re-stimulated with PMA/ionomycin (P+I) for 5 hr, lysed and immunoprecipitated with anti-c-Maf antibody. The immunoprecipitant was then probed with 4G10 or anti-c-Maf antibody. A fraction of un-precipitated extract was probed with anti-Tec and anti-Tubulin (the bottom two panels). Phosphorylation ratios of c-Maf are also shown.

Given the high expression of Tec kinases in T cells, we first examined if any of the Tec kinases was a tyrosine kinase of c-Maf. We co-expressed c-Maf with one of the Tec members in HEK 293T cells. All three Tec kinases were capable of auto-phosphorylation (S1 Fig). We detected a high level of tyrosine phosphorylated c-Maf only when Tec, but not Rlk or Itk, was co-expressed (Fig 1A). This effect was dependent on the kinase activity of Tec because co-expression of a kinase-dead Tec (Tec KD) did not lead to tyrosine phosphorylation of c-Maf. Moreover, both Tec and Tec KD, but not Rlk or Itk, co-immunoprecipitated with c-Maf (Fig 1A). In addition, the dominant Tec-induced phosphorylation sites were located at the N-terminal activation domain (residues 1–118) (Fig 1B). Trace phosphorylation was also detected in the hinge domain (residues 119–254). In contrast, no phosphorylation was detected in the C-terminal DNA binding domain (residues 255–370). We subsequently investigated whether Tec phosphorylated c-Maf at Tyr21/92/131. Indeed, mutation of Tyr21 and Tyr92 attenuated Tec-induced tyrosine phosphorylation and mutation of all three residues (Y3F) nearly completely ablated Tec-induced tyrosine phosphorylation, an effect very comparable to that seen with mutation of all 8 tyrosine residues of c-Maf (Y8F in Fig 1C). To determine whether Tec was able to phosphorylate endogenous c-Maf in primary T cells, T_H_2 cells were transduced with retrovirus expressing murine Tec or lentivirus expressing shRNA specific for Tec (shTec) (Fig 1D). We then examined the intensity of tyrosine phosphorylation of c-Maf 48 hours after transduction. Similar to the result obtained from HEK 293T cells, tyrosine phosphorylation of c-Maf was enhanced in activated T_H_2 cells by ectopically expressed Tec, whereas c-Maf tyrosine phosphorylation was reduced by knocking down Tec (Fig 1D).

**Fig 2:**
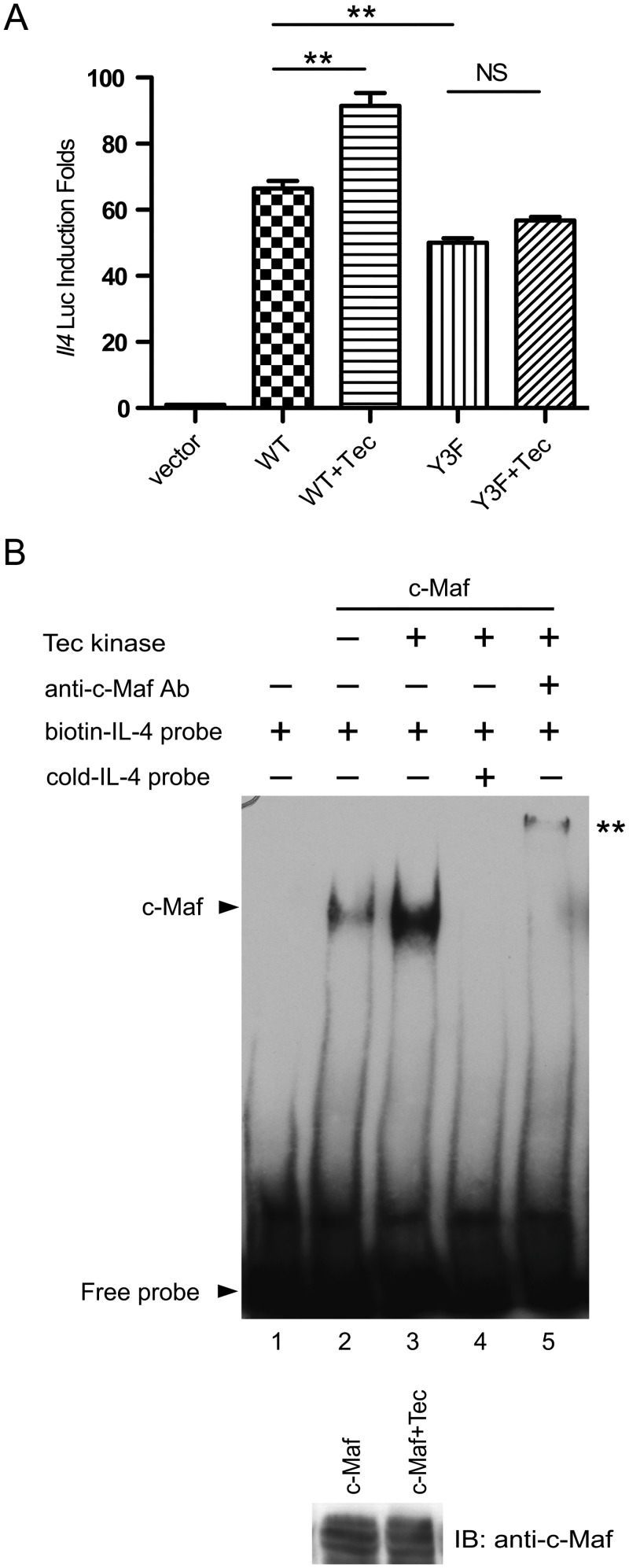
Tec enhances IL-4 promoter activity by facilitating the binding of c-Maf to the IL-4 promoter. (**A**) HEK 293T cells were transfected with pGL3-*Il4*-Luc and pRL-TK along with expression vectors for WT c-Maf, Y3F c-Maf, and/or Tec. The transfected cells were lysed after 24 hours and the luciferase activity was first normalized according to Materials and Methods and then against the value obtained with empty expression vector, which was arbitrarily set as 1. Each experiment was done in triplicate. The data shown are mean ± SEM from three independent experiments. ** *P*<0.01. NS stands for not significant. (**B**) Nuclear extracts were prepared from HEK 293T cells expressing c-Maf and Tec, and subjected to EMSA using a 33 bp biotinylated probe derived from the MARE element (-31 to -64) within the IL-4 promoter (biotin-IL-4 probe). Unlabeled IL-4 probe (cold-IL-4 probe) and anti-c-Maf were added to the indicated lanes. A fraction of the nuclear extract used in lanes 2 and 3 was analyzed with Western blotting using anti-c-Maf (the lower panel).

Tyrosine phosphorylation of c-Maf is required for optimal expression of IL-4 [10]. Therefore, we were interested in determining whether c-Maf-dependent IL-4 expression was enhanced by Tec. We found that co-expression of Tec increased c-Maf-induced IL-4 promoter-luciferase activity. Y3F c-Maf was less active than WT c-Maf and its activity was not further enhanced by Tec (Fig 2A). However, we were not able to detect a reproducible effect of Tec overexpression on endogenous IL-4 production by T_H_2 cells partly due to unexpected cytotoxic effects of exogenous Tec. Tyrosine phosphorylation of c-Maf facilitates its recruitment to the IL-4 promoter [10]. We found that overexpression of Tec enhanced the binding of c-Maf to the MARE site derived from the IL-4 promoter (Fig 2B). Thus, Tec is a tyrosine kinase of c-Maf and promotes IL-4 expression by facilitating the binding of c-Maf to the IL-4 promoter.

### Ptpn22 interacts with and dephosphorylates c-Maf

**Fig 3:**
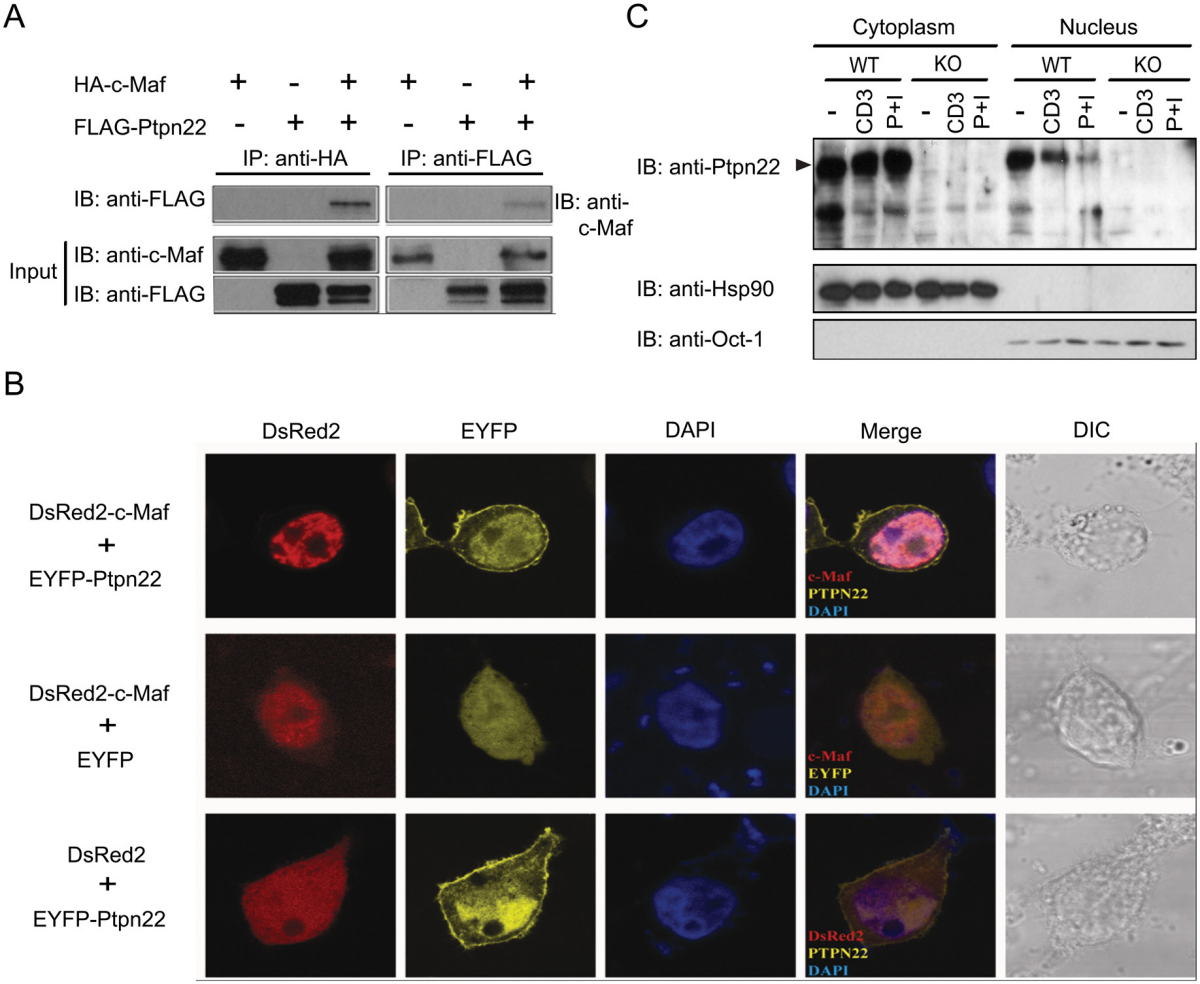
Ptpn22 is located in the nucleus of T cells and interacts with c-Maf. (**A**) HEK 293T cells were transfected with expression vectors encoding HA-c-Maf and/or FLAG-Ptpn22. Whole cell extract was harvested from the transfected cells 24 hours later and immunoprecipitated (IP) with anti-HA or anti-FLAG antibodies. The immunoprecipitant was then Immuno-blotted (IB) with anti-FLAG or anti-c-Maf antibodies. A fraction of the whole cell lysate was also analyzed directly with Western blot analysis using anti-c-Maf and anti-FLAG antibodies (Input). (**B**) HEK 293T cells were transfected with vectors expressing DsRed2-c-Maf or DsRed and EYFP-Ptpn22 or EYFP. Twenty-four hours later, the transfected cells were stained with DAPI and examined with confocal microscopy. DIC stands for differential interference contrast. (**C**) *In vitro* differentiated primary mouse T_H_2 cells were left unstimulated or re-stimulated with anti-CD3 antibody for 16 hours or with PMA and ionomycin (P + I) for 6 hours. Cytoplasmic and nuclear extract was separately prepared and analyzed with Western blotting using indicated antibodies. Hsp90 is a cytoplasmic protein whereas Oct1 represents nuclear protein.

In a parallel experiment, we used the hinge domain of c-Maf as bait to search for its interacting proteins. We obtained 42 positive colonies encoding fifteen candidates (Table 1). Three of the colonies encoded overlapping peptide fragments matching the PTP domain of Ptpn22 (S2 Fig). We subsequently expressed HA-tagged c-Maf and FLAG-tagged Ptpn22 in HEK 293T cells and confirmed their physical interaction with co-immunoprecipitation (Fig 3A). To further visualize the subcellular localization of c-Maf and Ptpn22, we co-expressed DsRed2-c-Maf and EYFP-Ptpn22 in HEK 293T cells. Expectedly, c-Maf was present exclusively in the nucleus. Ptpn22 was not only detected in the cytoplasmic membrane but also co-localized with c-Maf in the nucleus (Fig 3B). As c-Maf is located mainly in the nucleus of T cells, our data prompted us to examine whether Ptpn22 was also present in the nucleus of primary T cells. Indeed, a substantial fraction of Ptpn22 of primary T_H_ cells was located in the nucleus and the nuclear fraction of Ptpn22 was slightly reduced in response to stimulation with anti-CD3 or PMA/ionomycin (Fig 3C).

**Table 1:** List of fifteen c-Maf-interacting candidates obtained from yeast two-hybrid experiment.

Group	Name of Gene	Frequency
A	*Mus musculus* RIKEN cDNA 2010311D03 gene	5
B	*Mus musculus* protein tyrosine phosphatase, non-receptor type 22 (Ptpn22)	3
C	*Mus musculus* endothelial cell-specific molecule 1 (Esm1)	3
D	*Mus musculus* heat shock protein 8 (Hspa8)	3
E	*Mus musculus* golgi phosphoprotein 3-like	2
F	*Mus musculus* cleavage and polyadenylation specific factor 2 (Cpsf2)	1
G	*Mus musculus* S-adenosylhomocysteine hydrolase (Ahcy)	1
H	*Mus musculus* replication protein A1 (Rpa1)	1
I	*Mus musculus* protein kinase, cAMP dependent regulatory, type I, alpha	1
J	*Mus musculus* polymerase (RNA) II (DNA directed) polypeptide J	1
K	*Mus musculus* 0 day neonate head cDNA, clone:4833426G12	1
L	*Mus musculus* prosaposin (Psap)	1
M	*Mus musculus* CH-rich interacting match of PLAG1	1
N	*Mus musculus* BAC clone RP23-257F7	1
O	*Mus musculus* NADH dehydrogenase 6, mitochondrial	1

**Fig 4:**
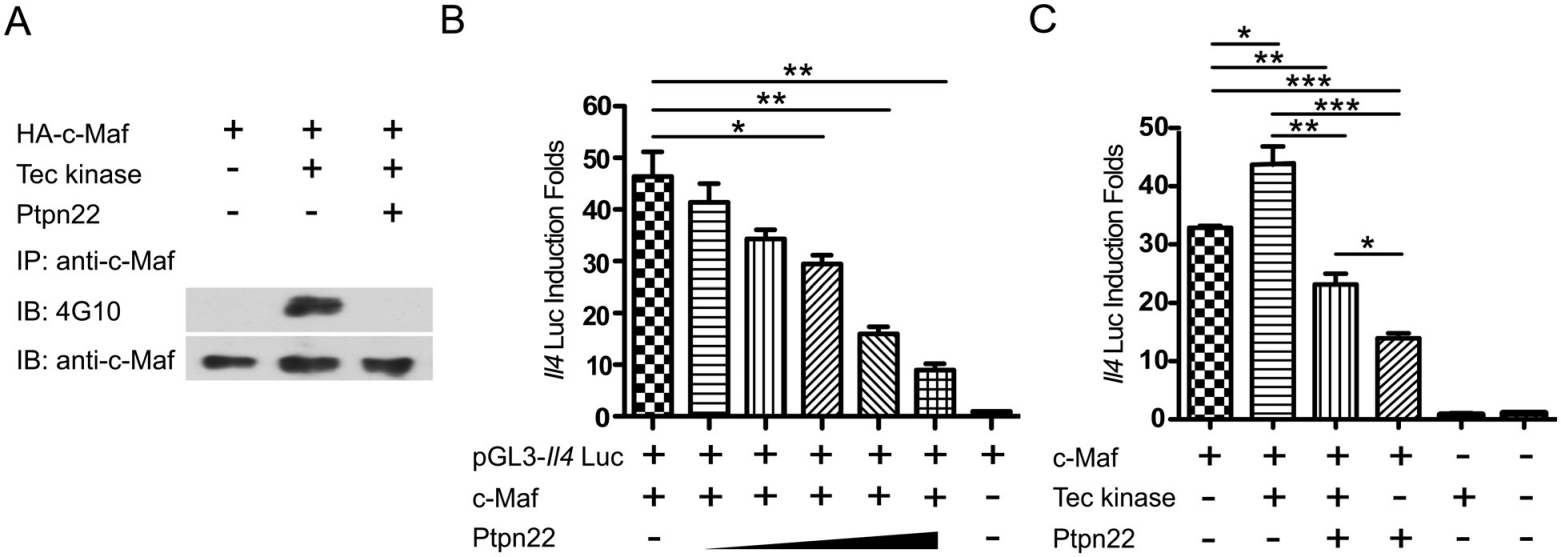
Ptpn22 attenuates tyrosine phosphorylation of c-Maf and reduces c-Maf-dependent transactivation of the IL-4 reporter. (**A**) HEK 293T cells were transfected with plasmids expressing HA-c-Maf, Tec and/or Ptpn22. The transfected cells were lysed and then immunoprecipitated with anti-c-Maf antibody. The immunoprecipitant was then examined with Western blotting using 4G10 or c-Maf antibody. (**B**) HEK 293T cells were transfected with pGL3-*Il4*-Luc, pRL-TK, along with vectors expressing c-Maf and escalating levels Ptpn22. Luciferase activity was quantified 24 hours later and normalized according to Materials and Methods. The normalized luciferase activity obtained from cells transfected with empty expression vector was arbitrarily set as 1. (**C**) HEK 293T cells were transfected with pGL3-*Il4*-Luc, pRL-TK together with vectors expressing WT c-Maf, Tec, and/or Ptpn22. The normalized luciferase activity was calculated as in (B). (B) and (C) were performed in triplicate. The data shown are mean ± SEM from three independent experiments. * *P*<0.05, ** *P*<0.01 and *** *P*<0.001.

**Fig 5:**
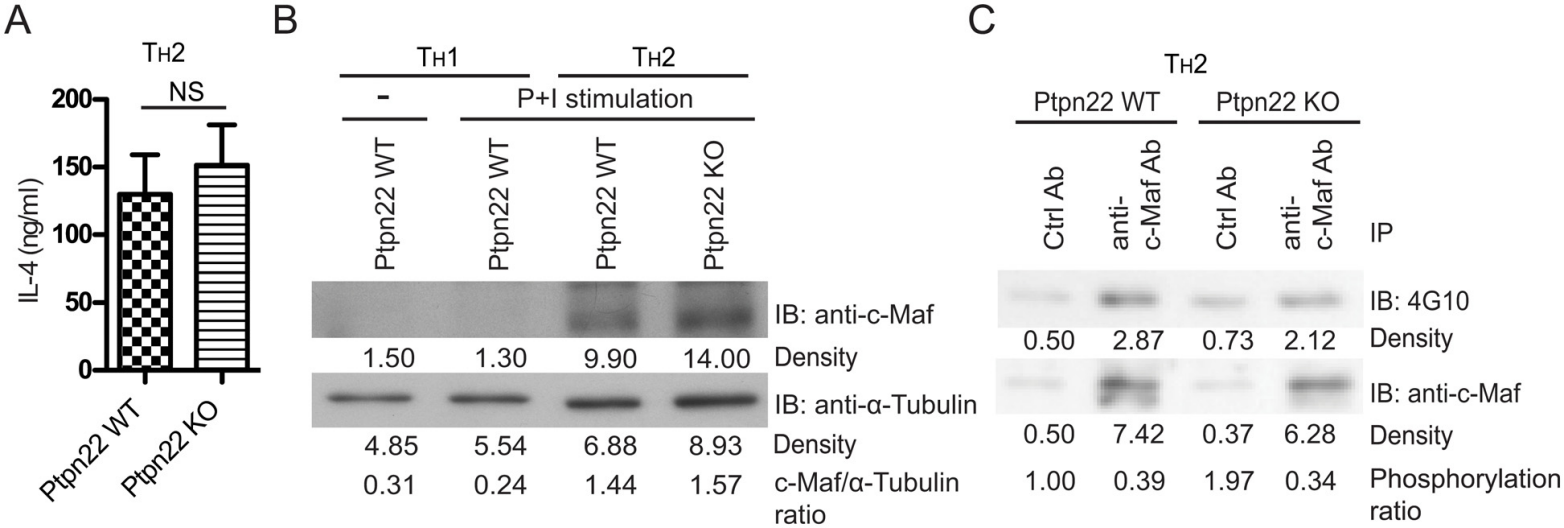
Deficiency of Ptpn22 has little impact on the level and tyrosine phosphorylation of c-Maf in T_H_2 cells. (**A**) Wild type (Ptpn22 WT) and Ptpn22 KO T_H_ cells were differentiated into T_H_2 cells. The differentiated cells were re-stimulated with anti-CD3 antibody and the production of IL-4 was measured with ELISA. Mean and SEM values were obtained from twelve independent samples (n = 12). NS stands for not significant. (**B**) Primary T_H_ cells were isolated from WT or Ptpn22 KO mice and skewed under T_H_1 or T_H_2 conditions. Cells were rested (-) or re-stimulated with PMA/ionomycin (P+I) for 4 hr. Cell extract was harvested and probed with anti-c-Maf Ab or anti-α–Tubulin antibody. The density of each band shown was quantified with ImageJ. The band with the lowest density on each panel was arbitrarily set as 1. The c-Maf/α–Tubulin ratio was calculated by dividing the relative density of c-Maf by that of α–Tubulin. (**C**) Cell extract from stimulated WT and Ptpn22KO T_H_2 cells described in (B) was also subjected to immunoprecipitation with anti-c-Maf antibody or control antibody. The immunoprecipitant was then probed with 4G10 or anti-c-Maf antibody. Phosphorylation ratios of c-Maf are also shown.

We subsequently postulated that Ptpn22 was a phosphatase of c-Maf. To test this hypothesis, we expressed WT c-Maf, Tec, and/or Ptpn22 in HEK 293T cells. As shown in Fig 4A, tyrosine phosphorylation of c-Maf was strongly enhanced by Tec and this Tec-induced tyrosine phosphorylation was completely mitigated by Ptpn22. In addition, Ptpn22 attenuated c-Maf-dependent IL-4 luciferase activity in a dose-dependent manner (Fig 4B) and counteracted the positive effect of Tec on reporter activity (Fig 4C). However, IL-4 production was very comparable between WT and Ptpn22KO T_H_2 cells (Fig 5A). In addition, the protein level and degree of tyrosine phosphorylation of c-Maf was normal in Ptpn22KO T_H_2 cells (Fig 5B and 5C), suggesting the presence of additional PTPs of c-Maf.

### C-Maf undergoes tyrosine phosphorylation in T_H_17 cells

**Fig 6:**
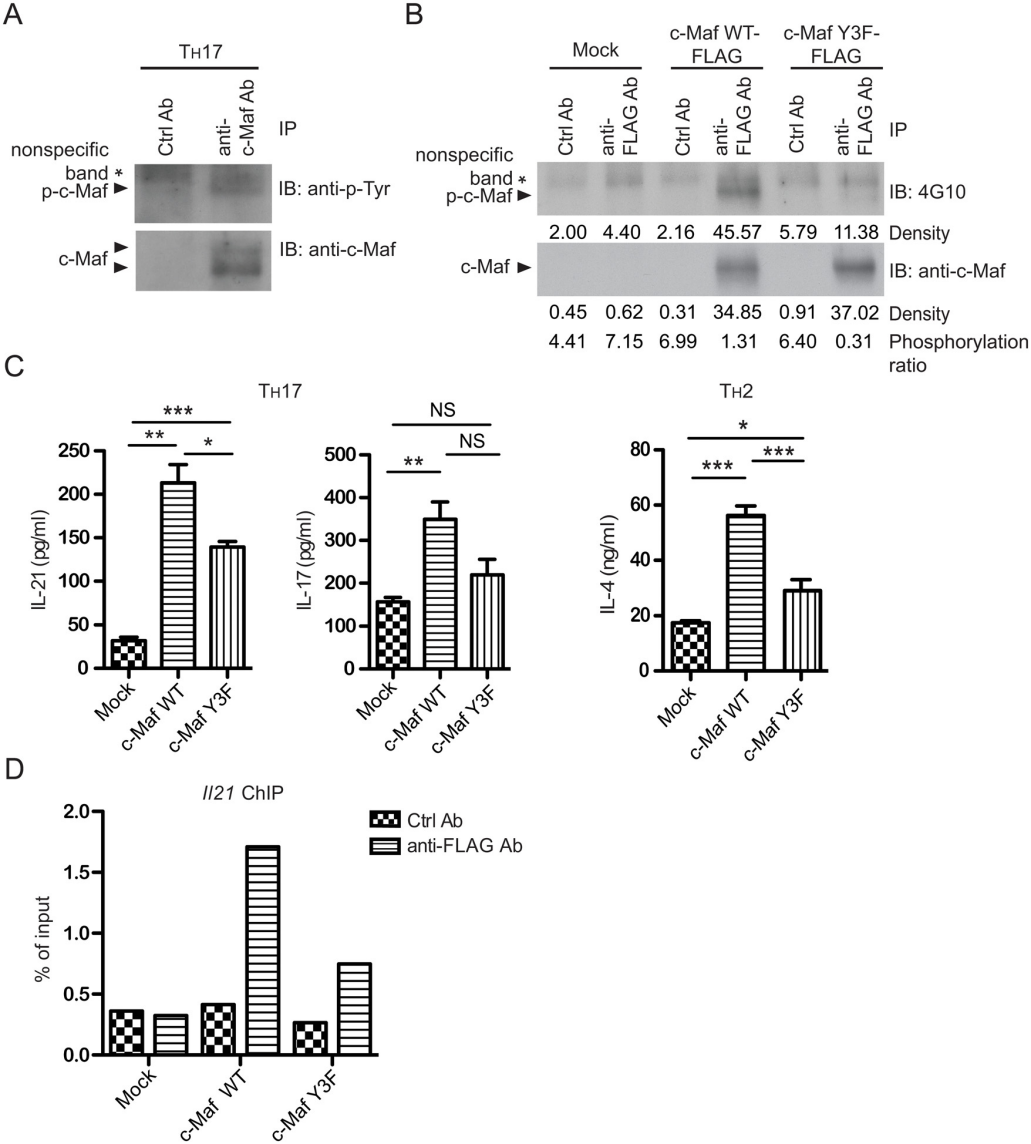
Tyrosine phosphorylation at Tyr21/92/131 of c-Maf is critical for optimal IL-21 production in T_H_17 cells. (**A**) Primary T_H_ cells were stimulated *in vitro* under T_H_17 skewing conditions for 72 hours. Cells were lysed and then immunoprecipitated with anti-c-Maf or control IgG. The immunoprecipitant was probed with anti-p-Tyr and anti-c-Maf antibodies. (**B**) Primary T_H_ cells were stimulated *in vitro* under T_H_17 skewing condition for 40 hours and transduced with retrovirus expressing c-Maf WT-FLAG or c-Maf Y3F-FLAG. Forty-eight hours later, transduced cells were stimulated with plate-bound anti-CD3 and soluble anti-CD28 antibodies for 24 hours. Cells were lysed and then immunoprecipitated with anti-c-Maf antibody. The immunoprecipitant was probed with 4G10 and anti-c-Maf antibodies. Phosphorylation ratios of c-Maf are also shown. (**C**) Primary T_H_ cells were stimulated *in vitro* under T_H_2 and T_H_17 skewing conditions for 40 hours and transduced with retrovirus expressing c-Maf WT-FLAG or Y3F-FLAG. Forty-eight hours later, transduced (GFP^+^) cells were sorted and re-stimulated with plate-bound anti-CD3 antibody for 24 hours. The levels of IL-4, IL-17 and IL-21 in supernatant were measured by ELISA in triplicate. The data shown are mean ± SEM from three independent experiments,* *P*<0.05 and *** *P*<0.001. (**D**) Retroviral transduced T_H_17 cells described in (C) were sorted according to GFP expression, re-stimulated with PMA and ionomycin for 1 hour, and subjected to ChIP using primers derived from the *Il21* promoter.

C-Maf is also expressed in T_H_17 cells [10]. To further examine whether c-Maf was also tyrosine phosphorylated in T_H_17 cells, primary CD4^+^ T cells were polarized into T_H_17 cells for 72 hours. Cell lysate of the polarized cells was then subjected to immunoprecipitation with either anti—c-Maf or control IgG and immunoblotted with anti-p-Tyr. As shown in Fig 6A, anti-c-Maf but not control IgG was able to precipitate a tyrosyl phospho-protein corresponding to the size of c-Maf. These data indicate that c-Maf is tyrosine phosphorylated in both T_H_2 and T_H_17 cells. Tyr21/92/131 are the dominant tyrosine phosphorylation sites of c-Maf in T_H_2 cells [10]. We then asked whether these three tyrosine residues were also the dominant phosphorylation sites of c-Maf in T_H_17 cells. We expressed FLAG-tagged WT or the Y3F mutant of c-Maf in primary T_H_17 cells. As shown in Fig 6B, the Y3F mutant was also resistant to tyrosine phosphorylation in primary T_H_17 cells. Our data collectively demonstrate that Tyr21/92/131 are the dominant tyrosine phosphorylation sites of c-Maf in both T_H_2 and T_H_17 cells.

### Phosphorylation of c-Maf at Tyr21/92/131 enhances the expression of IL-21 by facilitating its recruitment to the IL-21 promoter

One of the targets of c-Maf in T_H_17 cells is IL-21. We therefore examined whether tyrosine phosphorylation of c-Maf was necessary for maximal IL-21 production in T_H_17 cells. We expressed WT or Y3F c-Maf in primary T_H_17 cells. In agreement with previous report [4], forced expression of WT c-Maf induced the expression of IL-21 gene. However, Y3F c-Maf was only about 50% efficient as WT c-Maf (Fig 6C, the left panel). The impact of phosphorylation at Tyr21/92/131 on IL-21 production in T_H_17 cells was very comparable to that on IL-4 in T_H_2 cells (Fig 6C, the right panel). However, Y3F c-Maf did not affect the IL-17 production in T_H_17 cells, suggesting that tyrosine phosphorylation of c-Maf has minimal effect on T_H_17 cell differentiation (Fig 6C, the middle panel). We then examined whether tyrosine phosphorylation at Tyr21/92/131 also affected the binding of c-Maf to the IL-21 promoter in T_H_17 cells. We performed chromatin immunoprecipitation on primary T_H_17 cells expressing WT c-Maf or its Y3F mutant to compare their recruitment to the IL-21 promoter. The recruitment of Y3F c-Maf to the IL-21 promoter was approximately 60% less efficient than WT c-Maf (Fig 6D).

### Deficiency of Ptpn22 has little impact on the activity and tyrosine phosphorylation of c-Maf in T_H_17 cells

**Fig 7:**
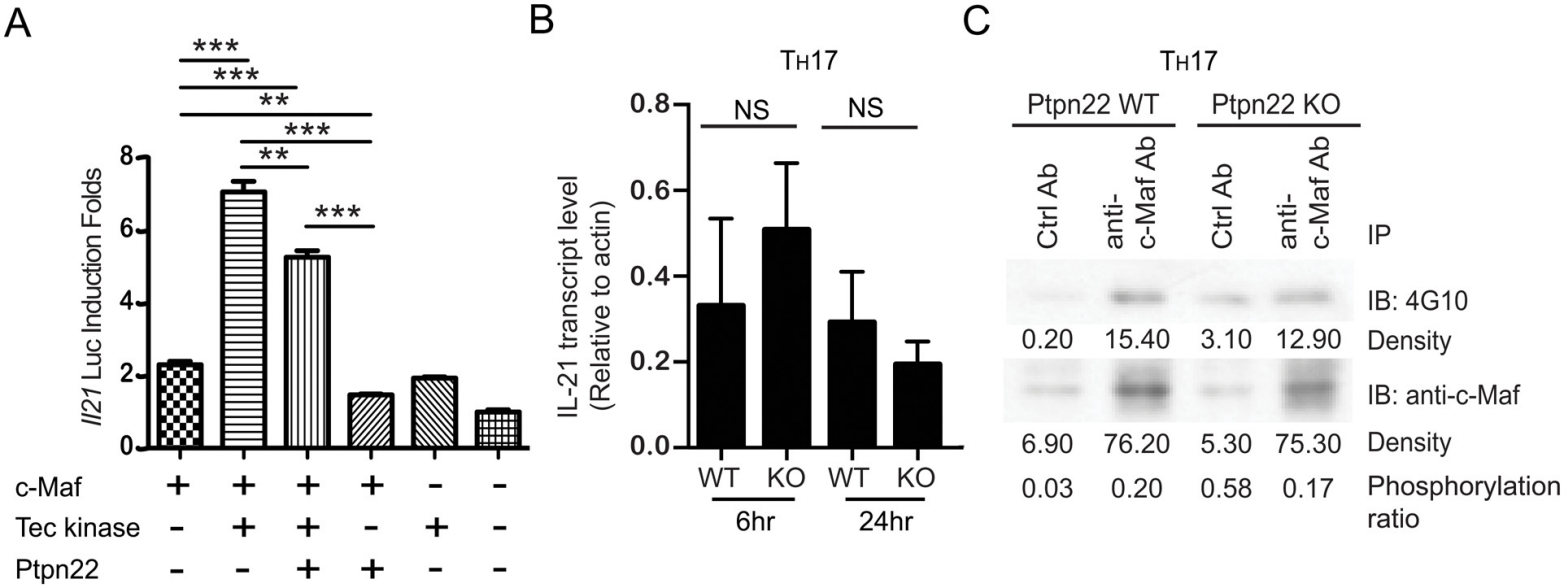
Normal protein level and degree of tyrosine phosphorylation of c-Maf in Ptpn22-deficient T_H_17 cells. (**A**) HEK 293T cells were transfected with pGL3-*Il21*-Luc, pRL-TK together with vectors expressing WT c-Maf, Tec, and/or Ptpn22. Luciferase activity was quantified 24 hours later and normalized according to Materials and Methods. The normalized luciferase activity obtained from cells transfected with empty expression vector was arbitrarily set as 1. The data shown are means ± SEM from three independent experiments. (**B & C**) Wild type (Ptpn22 WT) and Ptpn22 KO T_H_ cells were differentiated into T_H_17 cells. The differentiated cells were re-stimulated with anti-CD3 antibody for 6 hr or 24 hr and the expression of IL-21 gene was measured by qPCR analysis (**B**). Mean and SEM values were obtained from three independent experiments. NS stands for not significant. Whole cell extract was harvested after re-stimulation with anti-CD3 for 24 hr, and subjected to immunoprecipitation with anti-c-Maf antibody or control antibody. The immunoprecipitant was then probed with 4G10 or anti-c-Maf antibody (**C**). Phosphorylation ratios of c-Maf are also shown.

We subsequently examined whether Ptpn22 had any effect on c-Maf-dependent IL-21 promoter activity. As shown in Fig 7A, Tec enhanced c-Maf-dependent IL-21 luciferase activity whereas Ptpn22 counteracted the positive effect of Tec on reporter activity. However, IL-21 gene expression was normal in both WT and Ptpn22KO T_H_17 cells (Fig 7B). In addition, the protein level and degree of tyrosine phosphorylation of c-Maf was comparable in WT and Ptpn22KO T_H_17 cells (Fig 7C), suggesting the presence of additional PTPs of c-Maf.

## Discussion

We have identified a tyrosine kinase/phosphatase pair of c-Maf: Tec and Ptpn22, which reciprocally regulate the phosphorylation of c-Maf at Tyr21/92/131 and subsequently c-Maf-dependent transactivation of IL-4. We also demonstrated that phosphorylation at those three tyrosine residues was important for optimal expression of IL-21 in T_H_17 cells. A previous study has demonstrated that the expression of Tec is higher in T_H_2 cells than in T_H_1 cells [12]. In addition, the expression of Tec is low in naïve or resting T cells and is rapidly increased after TCR stimulation, a pattern coinciding with tyrosine phosphorylation of c-Maf during the activation of T cells. Our data also indicate that co-expression of Tec increased c-Maf-induced IL-4 promoter activity. However, Ellmeier *et al*. have shown that the percentage of IFNγ- and IL-4-producing CD44^hi^CD62L^-^ T cells or *in vitro* differentiated T_H_ cells was comparable between wild type and Tec-deficient mice. One explanation for the discrepancy is that T_H_ cells developing in the absence of Tec may acquire a compensatory mechanism to phosphorylate c-Maf. Alternatively, tyrosine phosphorylation of c-Maf is impaired in Tec-deficient T_H_ cells but the expression of IL-4 is compensated by other transcription factors, such as NFAT and c-Jun. It will be informative to examine the status of c-Maf tyrosine phosphorylation in Tec-deficient T_H_2 cells.

The observation that c-Maf tyrosine phosphorylation and the expression of IL-4 and IL-21 are intact in Ptpn22KO T_H_ cells strongly suggests that there are additional PTPs of c-Maf. Although the ternary structure of PTP domain is conserved among all PTPs, its surface is quite diverse and displays a wide spectrum of electrostatic potential, allowing the grouping of PTPs [31]. Phylogenetically, Ptpn22 is closely related to Ptpn12 and Ptpn18, both of which are expressed in T cells according to the Immgen database. It is possible that these two PTPs can also use c-Maf as a substrate and compensate for the loss of Ptpn22. Deficiency of Ptpn12 affected secondary T cell response presumably by acting on Pyk2 but it did not affect the development and primary response of T cells [32]. The role of Ptpn18 in regulating IL-4 expression is still unknown. It will be of great interest to determine whether Ptpn12, Ptpn18, and other PTPs also physically interact with c-Maf and regulate its tyrosine phosphorylation. An alternative explanation for the intact IL-4/IL-21 production in Ptpn22KO T_H_ cells is that the basal c-Maf activity and IL-4/IL-21 production in T_H_2/T_H_17 cells may be relatively independent of Ptpn22, which becomes influential only when Tec kinase expression/activity is enhanced. This alternative scenario raises the possibility that Tec may directly or indirectly recruit Ptpn22. This possibility is being investigated.

While we were able to co-immunoprecipitate c-Maf and Ptpn22 when both proteins were overexpressed in HEK 293T cells (Fig 3A), we were unable to demonstrate physical interaction between endogenous c-Maf and Ptpn22 in T_H_ cells. There are several possible explanations for this negative result. Both c-Maf and Ptpn22 are not abundant proteins and their interaction may be very transient. In addition, as discussed above, the interaction between c-Maf and Ptpn22 in primary T_H_ cells may occur only when Tec activity is induced. Thus, detailed analysis of the kinetics of c-Maf phosphorylation and expression of Tec and Ptpn22 in T_H_ cells under different stimulation conditions may eventually establish a definite role of Ptpn22 in regulating the phosphorylation and activity of c-Maf.

Ptpn22 is known to be present in the nucleus of macrophages. However, our data is the first to demonstrate that it is also expressed in the nucleus of primary T cells. Although deficiency of Ptpn22 has little impact on the expression of IL-4, the observation that Ptpn22 interacts with c-Maf in the nucleus indicates that Ptpn22 can modulate the function of T cells by acting on nuclear proteins in addition to regulating the strength of activation signals. Given the strong association of Ptpn22 in several human autoimmune diseases [24–26], identifying nuclear substrates of Ptpn22 may shed light on the pathogenesis of those diseases.

## Supporting Information

S1 Fig Recombinant Tec kinase family members (Tec, Itk and Rlk) are functionally active. The FLAG tagged-Tec kinase family plasmids, including Tec, Itk, Rlk, were transfected into HEK 293T cells. The cells were lysed after 24 hours and cell extract was subjected to immunoprecipitated (IP) with anti-FLAG antibody (M2). The immunoprecipitant was then probed with anti-p-Tyr, anti-FLAG, anti-Tec, anti-Rlk or anti-Itk antibody.
(PDF)

S2 Fig Schematic diagram of full-length Ptpn22 proteins and peptide fragments encoded in three prey clones obtained from yeast two-hybrid analysis. The PTP domain is shaded and marked.
(PDF)
