# Supplementary figures and images for: Reciprocal Regulation of C-Maf Tyrosine Phosphorylation by Tec and Ptpn22
Source: PLoS One. 2015 May 20;10(5):e0127617. doi: 10.1371/journal.pone.0127617 (PMC4439128; doi:10.1371/journal.pone.0127617)

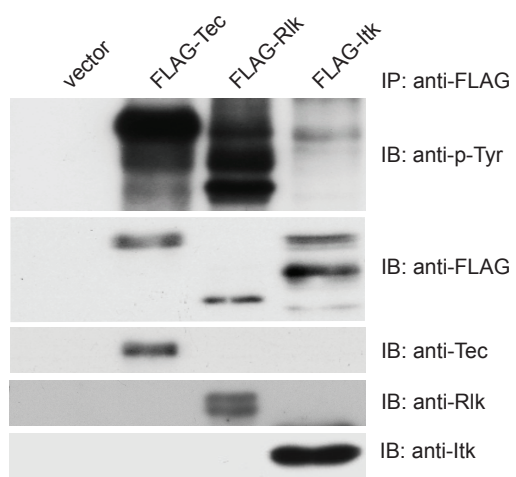

Supplement: S1 Fig — The FLAG tagged-Tec kinase family plasmids, including Tec, Itk, Rlk, were transfected into HEK 293T cells. The cells were lysed after 24 hours and cell extract was subjected to immunoprecipitated (IP) with anti-FLAG antibody (M2). The immunoprecipitant was then probed with anti-p-Tyr, anti-FLAG, anti-Tec, anti-Rlk or anti-Itk antibody. (PDF) [file pone.0127617.s001.pdf]

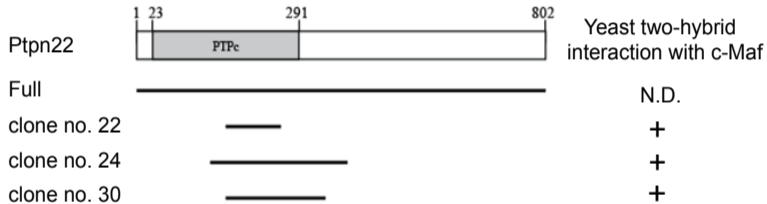

Supplement: S2 Fig — The PTP domain is shaded and marked. (PDF) [file pone.0127617.s002.pdf]
